# Relation of pulmonary diffusing capacity changes to HRCT chest and V/Q SPECT findings at short-term and intermediate follow-up after COVID-19: a prospective cohort study (The secure study)

**DOI:** 10.1007/s00259-025-07598-0

**Published:** 2025-10-30

**Authors:** Thora Wesenberg Helt, Rie Skovly Thomsen, Jan Christensen, Thomas Kromann Lund, Anna Kalhauge, Frederikke Rönsholt, Daria Podlekavera, Elisabeth Arndal, Anne-Mette Lebech, Ronan M. G. Berg, Terese L. Katzenstein, Jann Mortensen

**Affiliations:** 1 Department of Clinical Physiology and Nuclear Medicine, Copenhagen University Hospital – Rigshospitalet, Blegdamsvej 9, Copenhagen, 2100 Denmark; 2 Centre for Physical Activity Research, Copenhagen University Hospital – Rigshospitalet, Copenhagen, Denmark; 3 Department of Infectious Diseases, Copenhagen University Hospital – Rigshospitalet, Copenhagen, Denmark; 4 Department of Occupational Therapy and Physiotherapy, Copenhagen University Hospital – Rigshospitalet, Copenhagen, Denmark; 5 Department of Cardiology, Section for Lung Transplantation, Copenhagen University Hospital – Rigshospitalet, Copenhagen, Denmark; 6 Department of Radiology, Copenhagen University Hospital – Rigshospitalet, Copenhagen, Denmark; 7 Department of Otorhinolaryngology, Copenhagen University Hospital – Rigshospitalet, Copenhagen, Denmark; 8 Department of Clinical Medicine, Faculty of Health and Medical Sciences, University of Copenhagen, Copenhagen, Denmark; 9 Neurovascular Research Laboratory, Faculty of Life Sciences and Education, University of South Wales, Pontypridd, UK; 10 Department of Medicine, The National Hospital, Torshavn, Faroe Islands

**Keywords:** Pulmonary diffusing capacity, SARS-CoV-2, COVID-19, Long COVID

## Abstract

**Background:**

Several patients exhibit a severity-dependent reduced pulmonary diffusing capacity (D_LCOc_) following coronavirus disease 2019 (COVID-19) infection. This has been attributed to fibrosis-like restrictive lung disease, as shown by chest high-resolution computed tomography (HRCT), and concurrent ventilatory disturbances observed by ventilation-perfusion single-photon emission computed tomography (V/Q SPECT) imaging. The aim of this study was to investigate whether reductions in D_L,COc_ at short- and intermediate-term follow-up were associated with initial severity of COVID-19, and to which extend this was linked to the presence of fibrosis-like abnormalities on HRCT and ventilatory disturbances on V/Q SPECT.

**Methods:**

A total of 153 patients diagnosed with COVID-19 between March 2020 and March 2021 were included in the study. The patients underwent lung function testing, chest HRCT, and V/Q SPECT at short-term (5.6 months) follow-up. Individuals exhibiting any evidence of post-COVID-19 sequelae (*n* = 121) were also referred to intermediate follow-up (12.5 months).

**Results:**

At short-term follow-up, a severity dependent reduction in D_L,COc_ was observed, which was not evident at intermediate follow-up. At both short-term and intermediate follow-up, HRCT showed ground-glass opacity (GGO) and fibrosis-like abnormalities related to disease severity. Most patients had V/Q defects mainly with ventilatory abnormalities, including both matched and inversely matched defects at both follow-up times.

**Conclusion:**

The severity-dependent reduction in D_L,COc_ at short-term follow-up, associated with restrictive lung function pattern, GGO and fibrosis on HRCT, and ventilatory disturbances on V/Q SPECT, showed spontaneous recovery by intermediate follow-up. However, the restrictive ventilatory disturbances and associated morphological changes persisted.

**Supplementary Information:**

The online version contains supplementary material available at 10.1007/s00259-025-07598-0.

## Introduction

The pulmonary symptoms of coronavirus disease 2019 (COVID-19) that persist beyond the immediate severe acute respiratory syndrome coronavirus 2 (SARS-CoV-2) infection include common complaints such as breathlessness, chest pain, and fatigue [1]. These symptoms, collectively termed ‘long COVID,’ are linked to a severity-dependent reduction in pulmonary diffusing capacity, as reported in several studies [2–5]. Morphologically, this has been attributed to a fibrosis-like restrictive lung disease as shown by chest high-resolution computed tomography (HRCT) [6–8], with concomitant ventilatory disturbances as shown by ventilation-perfusion single-photon emission computed tomography (V/Q SPECT) imaging [3].

The post-COVID reduction in diffusing capacity appears to be more prevalent at short-term follow-up, i.e., 3–12 months, compared to intermediate follow-up, i.e., 12–18 months, and normalises in most cases [7, 9, 10]. Nonetheless, residual radiological abnormalities on chest CT scans are often observed, most commonly in the form of ground-glass opacities (GGO), interlobular septal thickening, and reticulations [3, 6, 8, 11–13]. Whether this apparent dissociation between lung function and morphological changes on CT can be conclusively established remains uncertain, primarily due to a paucity of serial assessments of diffusing capacity in conjunction with chest CT imaging after COVID-19 beyond short-term follow-up times [7, 8].

The present Danish SECURe (Sequelae of COVID-19 at Copenhagen University Hospital – Rigshospitalet) study is a prospective cohort study aimed at monitoring the severity and duration of post-COVID complications through comprehensive clinical, physiological, and chest imaging assessments in both previously hospitalised and non-hospitalised COVID-19 patients. In the current study, we investigated: (1) the extent to which the severity of diffusing capacity reduction at short- and intermediate-term follow-up was associated with the initial severity of COVID-19; (2) whether this was linked to the presence of fibrosis-like morphological abnormalities on HRCT and ventilatory disturbances on V/Q SPECT at both short-term and intermediate follow-up; and (3) mucociliary function, assessed by nasal saccharin clearance, at both short-term and intermediate follow-up. The latter was included as it has previously been reported to be abnormal in many cases post-COVID [14] and may adversely affect lung function.

We hypothesised that reductions in diffusing capacity and HRCT abnormalities would be associated with the severity of the initial COVID-19 infection at short-term follow-up. Furthermore, we anticipated that diffusing capacity would recover spontaneously to a greater extent than structural abnormalities on HRCT, such that any association with initial COVID-19 severity would persist only for HRCT findings.

## Methods

### Study design and setting

**Fig. 1:**
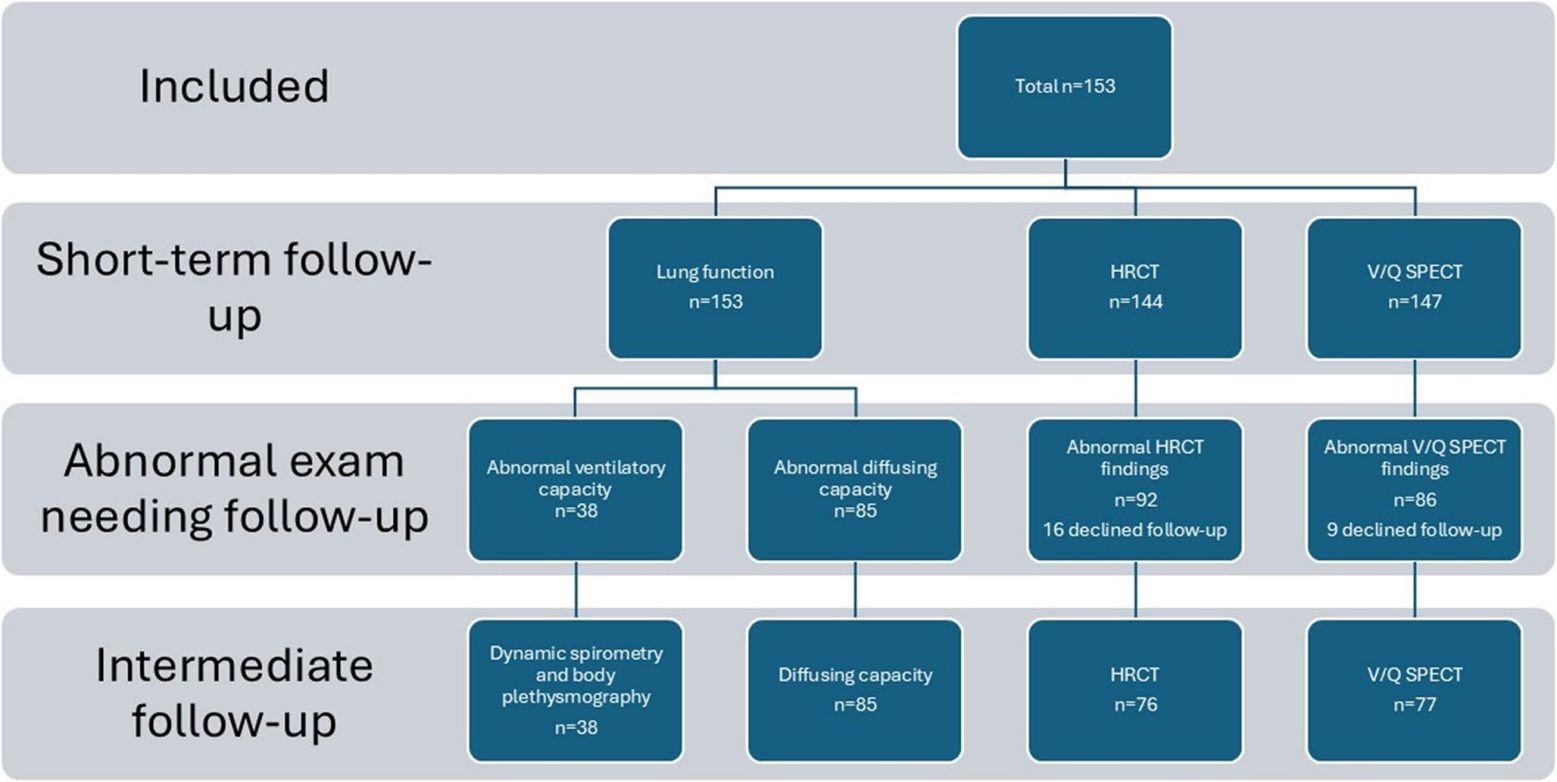
Study overview. Number of study participants from the SECURe study that underwent lung function testing, chest high-resolution computed tomography (HRCT), and ventilation-perfusion single-photon emission computed tomography (V/Q SPECT) at short-term and intermediate follow-up after COVID-19

The SECURe study was a prospective cohort investigation of individuals with SARS-CoV-2 infection confirmed by polymerase chain reaction testing using oral or nasopharyngeal swabs, conducted between March 1, 2020, and March 31, 2021. Participants underwent comprehensive evaluations, including assessments of symptom severity, lung function tests, and imaging studies such as V/Q SPECT and HRCT. For hospitalised patients, the initial SECURe study visit was scheduled approximately three months post-discharge, while for non-hospitalised participants, it occurred around three months after testing positive for SARS-CoV-2; both were classified as short-term follow-up in the present study. According to the SECURe protocol, participants with findings indicative of COVID-19 sequelae were referred for re-assessments at approximately 12 months, which was classified as intermediate follow-up. Therefore, intermediate follow-up was only performed in those with abnormal findings at short-term follow-up on either spirometry, diffusing capacity, V/Q SPECT or HRCT, and not all examinations were performed in each participant. A diagram representing the participants undergoing each examination can be found in Fig. 1.

The study was conducted collaboratively across several departments at Rigshospitalet, a specialised University Hospital in Denmark primarily offering tertiary healthcare services. In Denmark, medical care is universally accessible through a publicly funded healthcare system. The monitoring protocol was informed by studies from the 2002–2003 SARS outbreak in Hong Kong [15] and early data from China [13, 16]. Treatment strategies for COVID-19 in Denmark evolved during the study; for example, steroid use, which was initially not standard, became routine from June 2020 following updated guidelines [17]. Similarly, remdesivir usage increased during the study period, with patients from the early phase of the epidemic participating in clinical trials before the drug became widely available from August 2020 [18].

### Study participants

All consecutive COVID-19 patients admitted to Rigshospitalet between March 2020 and March 2021 were invited to participate in the study. Additionally, individuals with confirmed SARS-CoV-2 infection who did not require hospitalisation were offered inclusion, with the aim of enrolling two hundred participants, at least two-thirds of whom were hospitalised. Age was determined as the difference between the visit date and the participant’s birth date. Severity status was categorised as asymptomatic, mild, moderate, severe, or critical, based on NIH guidelines and definitions [19, 20]. Findings from lung function tests, HRCT, and V/Q SPECT at short-term follow-up for the first 67 participants have been reported in a previous publication [3]. Additional studies based on this cohort, including dual-gas pulmonary single-breath capacity for carbon monoxide and nitric oxide [5], respiratory muscle strength [21], and olfactory and gustatory outcomes [22], have also been published elsewhere.

### Recruitment

At discharge and/or during a one-month post-discharge telephone consultation, patients were informed about the study. This telephone consultation was routinely offered to all COVID-19 patients admitted to the Department of Infectious Diseases at Rigshospitalet. Non-hospitalised individuals were also invited to participate. During the initial wave of the pandemic, non-admitted participants were identified through the Rigshospitalet-affiliated testing site. However, following changes in the strategy for reporting positive test results, subsequent recruitment relied on word-of-mouth, resulting in most non-admitted participants being healthcare personnel. Recruitment was concluded at the end of March 2021 due to a significant decrease in SARS-CoV-2 transmission rates in Denmark and the closure of the dedicated COVID-19 ward at Rigshospitalet.

### Data sources

The following information was extracted from the participant’s electronic health record (Sundhedsplatformen, Epic SystemsCorporation, Wisconsin, USA); age, sex, result of standard blood tests (Haemoglobin, ferritin, platelets, leucocytes, neutrophils, CRP, D-dimer, ALAT, creatinine), co-morbidity status (Charlson Comorbidity Index, CCI [23]), date of testing SARS-CoV-2 positive, initial symptoms of COVID-19 and duration hereof prior to admission, treatment during hospitalisation including maximal oxygen demand, intensive care unit (ICU) admission, mechanical ventilation and/or extra-corporal membrane oxygenation and duration thereof, as well as total duration of hospitalisation. To assess symptom severity during follow-up, the chronic obstructive pulmonary disease (COPD) assessment test (CAT) was used, which is a questionnaire designed to assess the impact of COPD on everyday life activities and enables the evaluation of changes over time [24].

### Lung function testing

Lung function assessments included dynamic spirometry, body plethysmography, and single-breath measurement of the haemoglobin-corrected pulmonary diffusing capacity for carbon monoxide (D_L,COc_) and were conducted in accordance with ERS/ATS guidelines [25, 26]. Testing was performed at a specialised lung function facility within the Department of Clinical Physiology and Nuclear Medicine at Rigshospitalet, using a MasterScreen device (Vyaire Medical, Würzburg, Germany). Prior to testing, participants’ standing height (to the nearest 1 mm), weight (to the nearest 100 g), and haemoglobin (Hb) levels (to the nearest 0.1 mmol/L) were recorded. Hb was measured using capillary blood samples with a HemoCue^®^ analyser (Hb 201+; HemoCue, Denmark).

The variables analysed in this study include forced expiratory volume in the first second (FEV_1_), forced vital capacity (FVC), the FEV_1_/FVC ratio, total lung capacity (TLC), residual volume (RV), the RV/TLC ratio, D_L,COc,_ and the diffusion coefficient for carbon monoxide (K_CO_). These values were reported both as raw measurements and as percentages of predicted values, normalised for height, sex, and age based on ERS reference standards. Participants with FEV_1_/FVC ratios or TLC values below the lower limit of normal were classified as having obstructive or restrictive ventilatory patterns, respectively [27, 28].

### HRCT chest scan

The HRCT chest scan protocol included two consecutive acquisitions, with patients scanned in the supine position following a breath-hold at full inspiration and full expiration. The protocol used the following parameters: 130 kV, quality reference of 120 mAs, cranio-caudal or caudo-cranial direction, 16 × 1.2 mm acquisition, 1.0 pitch, 0.6-second rotation time, and a slice thickness of 1.5 mm. All HRCT scans were reviewed in consensus by two experienced diagnostic readers (TKL [pulmonologist] and AK [radiologist]). Each scan was divided into six zones (three on each side) for evaluation of: (1) GGO; (2) pulmonary fibrosis, identified by reticulation, traction, and bronchiolectasis; and (3) honeycombing. For these findings, the extent in each zone was scored on a scale from 0 to 4: 0 = none (normal), 1 = 1–25%, 2 = 26–50%, 3 = 51–75%, and 4 = >75% of the zone [29]. Additionally, the scans were assessed for emphysema, nodules, tracheo- or bronchomalacia, bronchiectasis, and air trapping.

### V/Q SPECT

V/Q SPECT was performed on a dual-head 16-slice CT Siemens Intevo BOLD SPECT/CT scanner (Siemens, Erlangen, Germany). A simultaneous dual-isotope technique was applied, utilising ^81m^Kr as the ventilation tracer and [^99m^Tc]Tc-macroaggregated albumin as the perfusion tracer. The imaging protocol included a 128 × 128 matrix, 128 angles, a MELP collimator, a total acquisition time of 11 min, and iterative Flash 3D reconstruction (3D OSEM with 4 iterations, 16 subsets, and a 12 mm FWHM Gaussian filter). A low-dose CT scan was obtained during free breathing (130 kV, quality reference 30 mAs, acquisition 16 × 0.6 mm, 0.6-second rotation time, 1.2 pitch, 3 mm slice thickness, cranio-caudal direction) for attenuation correction.

Interpretation followed the criteria’s recommended by the European Association of Nuclear Medicine [30]. Perfusion and ventilation defects were visually identified, localised, and classified as mismatched (defect in perfusion only), matched (combined perfusion and ventilation defect), or inversely mismatched (defect in ventilation only), and were further characterised as subsegmental or segmental. Ventilation defects classified as matched or inversely mismatched were considered ventilatory abnormalities regardless of HRCT chest findings. Mismatched perfusion defects without accompanying signs of GGO, reticulation, or fibrosis in the corresponding area on HRCT were categorised as vascular abnormalities. Two experienced pulmonary nuclear medicine specialists (RMGB and JM) independently reviewed all scans, with discrepancies resolved through consensus. Most reading were in concordance between the two readers and discrepancies resolved through consensus were found in 21% of the scans.

### Nasal clearance

Nasal mucociliary clearance of saccharin was performed as described elsewhere [31]. Briefly, a sodium saccharin particle, at least 1 mm in size, was placed under direct visualisation on the medial surface approximately 1 cm posterior to the head of the inferior nasal turbinate in both nostrils. Pre-taste verification was not conducted to avoid potential alterations in the results, as the saccharin taste may persist for up to four hours. Participants were asked to report as soon as they detected a sweet taste. The elapsed time was recorded to the nearest minute, and the test was deemed complete at that point. If no sweet taste was reported within 30 min, a time of >30 min was noted.

### Statistical analyses

All data were entered into REDcap (v.13.7.14 © 2024 Vanderbuilt University, TN, USA). All statistical analyses were performed using STATA (v. 18, StataCorp, Stata Statistical Software, College Station, TX, USA). Clinical characteristics (age, sex, CCI, CAT score, comorbidity, and anticoagulation), time from positive test and discharge to follow-up, HRCT, V/Q SPECT, lung function, and blood samples were summarized as n (percentage), mean (SD) for normally distributed variables or median [interquartile range] for non-normally distributed variables based on visual inspection of histograms and probability plots. The differences between severity groups were assessed using Fisher’s exact test for dichotomous and categorical variables, one-way ANOVA for normally distributed variables, and Kruskal-Wallis test for non-normally distributed variables. If a difference was found, bivariate comparisons with Holm-Sidák correction for multiple comparisons were made using Dunn’s test [32–34]. For the normally distributed variables, difference over time within COVID-19 severity groups were calculated using Student’s paired t-test. For the non-normally distributed variables, bootstrap with bias correction was used to calculate 95% confidence intervals [35, 36]. Univariate least square linear regression models were used to assess the association between CAT score, HRCT findings, or V/Q defects with D_L,COc_. Multiple logistic regression models were used to assess the association between V/Q defects, HRCT findings or D_L,COc_ with severity group, age, and sex. For all data, a two-sided *p* < 0.05 was considered statistically significant.

## Results

A total of 153 individuals were included in the study, all of whom underwent short-term follow-up at median 5.6 [4.7–6.6] months after diagnosis, while 121 underwent intermediate follow-up at median 12.5 [12.1–13.1] months after diagnosis. For a demographic description of the cohort, see Table 1. Higher clinical severity was associated with increased age, male sex, a greater burden of pre-COVID comorbidities, and anticoagulation treatment. Seventy-nine participants received low molecular weight heparin in prophylactic doses, while three received it in therapeutic doses, and six received a novel oral anticoagulant.

**Table 1:** Characteristics of patients with COVID-19 (*n* = 153) for each severity group

Parameters	*n*	All	Asymptomatic (*n* = 1)	Mild (*n* = 38)	Moderate (*n* = 23)	Severe (*n* = 69)	Critical (*n* = 22)	Between group *p*-value
Age at diagnosis, years	153	54.6 (15.4)	43	44.9 (12.8)	48.5 (19.0)	60.8 (13.4)	58.7 (11.9)	< 0.001^A^
Sex, male	153	84 (55%)	0 (0%)	12 (32%)	8 (35%)	48 (70%)	16 (73%)	< 0.001^A^
CCI	153	2 [1; >3]	1	1 [0; 2]	1 [0; 2]	3 [2; <3]	3 [2; <3]	< 0.001^A^
CAT score	153	6 [2; 10]	10	6 [2; 10]	6 [2; 12]	5 [1; 9]	7 [4; 10]	0.49
Co-morbidity COPD Ischaemic heart disease Diabetes Other	153	84 (55%) 7 (8%) 23 (27%) 20 (24%) 67 (80%)	1 (100%)	12 (32%)	12 (52%)	40 (58%)	19 (86%)	< 0.001^B^
Anticoagulation treatment	153	93 (61%)	0 (0%)	8 (21%)	10 (43%)	54 (78%)	21 (95%)	< 0.001^A^
Before diagnosis		8 (5%)	0 (0%)	2 (5%)	1 (4%)	5 (7%)	0 (0%)	
After diagnosis		85 (56%)	0 (0%)	6 (16%)	9 (39%)	49 (71%)	21 (95%)	
Time from positive SARS CoV-2 PCR test to first follow-up, days	153	170 [143; 200]	129	183 [148; 216]	156 [104; 204]	175 [156; 196]	155 [141; 215]	0.47
Time from positive SARS CoV-2 PCR test to second follow-up, days	121	380 [368; 398]	353	369 [362; 375]	370 [365; 384]	380 [367; 394]	405 [394; 429]	< 0.001^C^
Time from discharge to first follow-up, days	124	157 [105; 186]	-	181 [140; 196]	141 [96; 199]	166 [141; 185]	121 [95; 164]	0.053

Data are expressed as *n* (%), mean (SD) or median [interquartile range] as appropriate. Abbreviations: CCI: Charlson Comorbidity Index, CAT score: chronic obstructive pulmonary disease assessment test, COPD: chronic obstructive pulmonary disease. (A) Mild and moderate differ from severe and critical; (B) Critical differs from mild, moderate, and severe and mild differs from severe; (C) Critical differs from asymptomatic, mild, moderate, and severe

### Lung function testing

Lung function parameters according to clinical severity groups are summarised in Online Resource 1, with differences between short-term and intermediate follow-up presented in Online Resource 2. At short-term follow-up, lower FVC, TLC, RV, and D_L,COc_ were observed, typically reflecting a restrictive ventilatory pattern associated with clinical severity. Improvements were noted in FEV_1_, FVC, and D_L,COc_ from short-term to intermediate follow-up with these pulmonary function metrics no longer being related to clinical severity at intermediate follow-up. Nevertheless, a restrictive pattern was still the most common finding. Only the reduction in RV remained associated with clinical severity.

### HRCT chest scan

HRCT findings according to clinical severity groups are summarised in Table 2. At both short- and intermediate-term follow-up, the most common HRCT finding was GGO (60% of individuals at short-term and 88% of the individuals also investigated at intermediate follow-up), with its frequency depending on clinical severity. Other findings associated with clinical severity at both follow-up times included fibrosis and bronchiectasis.

**Table 2:** HRCT findings in patients at short-term and intermediate follow-up after COVID-19 (*n* = 144;76) and differences between severity groups

Parameter	*n*	All (*n* = 144;76)	Asymptomatic (*n* = 1;0)	Mild (*n* = 36;4)	Moderate (*n* = 21;12)	Severe (*n* = 65;44)	Critical (*n* = 21;16)	Between group *p*-value
Any GGO								
short-term follow-up	144	87 (60%)	0 (0%)	6 (17%)	12 (57%)	49 (75%)	20 (95%)	< 0.001^J^
intermediate follow-up	76	67 (88%)	-	3 (75%)	8 (67%)	40 (91%)	16 (100%)	0.029
Only GGO								
short-term follow-up	144	26 (18%)	0 (0%)	1 (3%)	8 (38%)	16 (25%)	1 (5%)	< 0.001^L^
intermediate follow-up	76	19 (25%)	-	2 (50%)	4 (33%)	11 (25%)	2 (13%)	0.32
> 25% GGO*								
short-term follow-up	144	41 (28%)	0 (0%)	0 (0%)	1 (5%)	24 (37%)	16 (76%)	< 0.001^K^
intermediate follow-up	76	23 (30%)	-	0 (0%)	0 (0%)	13 (30%)	10 (63%)	0.001^M^
Fibrosis (PF and/or HC)								
short-term follow-up	144	61 (42%)	0 (0%)	5 (14%)	4 (19%)	33 (51%)	19 (90%)	< 0.001^O^
intermediate follow-up	76	48 (63%)	-	1 (25%)	4 (33%)	29 (66%)	14 (88%)	0.008^N^
Air trapping								
short-term follow-up	144	16 (11%)	0 (%)	3 (8%)	0 (0%)	8 (12%)	5 (24%)	0.15
intermediate follow-up	76	13 (17%)	-	1 (25%)	1 (8%)	8 (18%)	3 (19%)	0.85
Bronchiectasis								
short-term follow-up	144	38 (26%)	0 (0%)	6 (17%)	4 (19%)	16 (25%)	12 (57%)	0.014^F^
intermediate follow-up	76	27 (36%)	-	2 (50%)	0 (0%)	15 (34%)	10 (63%)	0.003^N^
Tracheobronchomalacia								
short-term follow-up	144	8 (6%)	0 (0%)	1 (3%)	0 (0%)	5 (8%)	2 (10%)	0.51
intermediate follow-up	76	3 (4%)	-	0 (0%)	0 (0%)	2 (5%)	1 (6%)	1.00
Other**								
short-term follow-up	144	68 (47%)	0 (0%)	13 (36%)	8 (38%)	36 (55%)	11 (52%)	0.23
intermediate follow-up	76	42 (55%)	-	1 (25%)	7 (58%)	26 (59%)	8 (50%)	0.59

Data are expressed as *n* (%). Abbreviations: GGO: Ground –glass opacities, HC: honeycombing, PF: pulmonary fibrosis

*In more than one zone; **Noduli, enlarged pulmonary trunk, emphysema

F) Critical differs from mild, moderate, and severe; J) Mild differs from moderate, severe, and critical and moderate differs from critical; K) Critical differs from asymptomatic, mild, moderate, and severe and severe differs from mild and moderate; L) Mild differs from moderate and severe and moderate differs from critical; M) Critical differs from mild, moderate, and severe and moderate differs from severe; N) Critical differs from moderate; O) Critical differs from mild, moderate, and severe and severe differs from mild and moderate

### V/Q SPECT

**Fig. 2:**
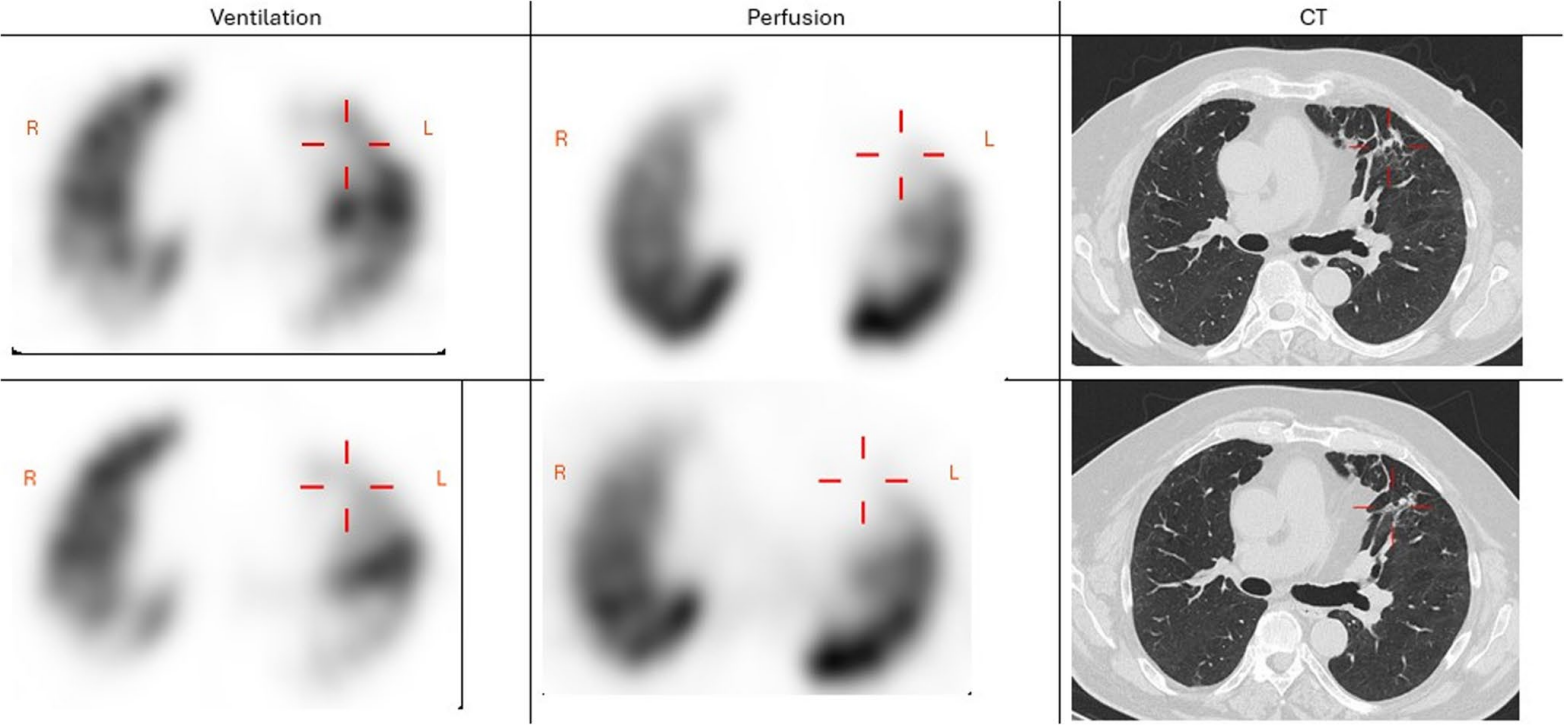
Chest imaging findings at short-term and intermediate follow-up in a 71-year-old male. Ventilation-perfusion single-photon emission computed tomography (V/Q SPECT) with high-resolution computed tomography (HRCT) shows a subsegmental mismatched perfusion defect with slightly reduced ventilation in the left segment 3 (red crosses on axial projections), both at short-term follow-up (6 months after COVID-19; upper row) and at intermediate follow-up (13 months after COVID-19; lower row). At both timepoints, HRCT shows reticulation, fibrosis and emphysema, but these are less pronounced at intermediate follow-up, indicating that the mismatched perfusion defect is caused by fibrosis rather than thromboembolic disease. Also, hypoventilation with inverse mismatched ventilation defects are seen in the posterior parts of both lungs at both short-term and intermediate follow-up. Overall, the chest imaging findings are compatible with a primary ventilatory disturbances due to fibrosis-like changes in the lung. Accordingly, lung function testing showed moderate restriction with a total lung capacity that was 67% of predicted at both time points, while the forced vital capacity improved slightly from 74% to 80% of predicted, and the pulmonary diffusing capacity improved from 59% to 67% of predicted

V/Q SPECT findings according to clinical severity at short-term and intermediate follow-up are summarised in Table 3. Almost all participants (87% of individuals that underwent short-term, and 83% of the individuals that also underwent intermediate follow-up) exhibited at least one type of V/Q defect. Ventilatory abnormalities were predominantly observed (64% at short-term and 61% at intermediate follow-up) with concomitant matched or inversely mismatched V/Q defects, with increasing frequency in higher clinical severity groups. In contrast, vascular abnormalities were relatively rare (16% at short-term and 13% at intermediate follow-up) and unrelated to clinical severity, despite frequent mismatched perfusion defects. Most such defects were not interpreted as primary pulmonary vascular disease, such as pulmonary embolism, as they were typically associated with HRCT findings, particularly signs of fibrosis. An example of V/Q SPECT and HRCT are shown in Fig. 2.

**Table 3:** V/Q SPECT findings in patients at short-term and intermediate follow-up after COVID-19 (*n* = 147;77) and differences between severity groups

Parameter	*n*	All (*n* = 147;77)	Asymptomatic (*n* = 1;1)	Mild (*n* = 36;13)	Moderate (*n* = 21;14)	Severe (*n* = 67;34)	Critical (*n* = 22;15)	Between group *p*-value
Ventilatory abnormality								
short-term follow-up	147	94 (64%)	1 (100%)	13 (36%)	14 (67%)	45 (67%)	21 (95%)	< 0.001^D^
intermediate follow-up	76	46 (61%)	0 (0%)	4 (33%)	8 (57%)	22 (65%)	12 (80%)	0.082
Vascular abnormality								
short-term follow-up	147	24 (16%)	0 (0%)	4 (11%)	6 (29%)	13 (19%)	1 (5%)	0.20
intermediate follow-up	77	10 (13%)	0 (0%)	2 (15%)	2 (14%)	6 (18%)	0 (0%)	0.49
V/Q defects at short-term follow-up	146	127 (87%)	1 (100%)	26 (72%)	21 (100%)	59 (89%)	20 (91%)	0.029^S^
Subsegmental, total	116	505	4	83	92	226	100	
Subsegmental, ratio		4.4	4	3.5	4.6	4.3	5.3	
Segmental, total	62	163	0	16	34	85	28	
V/Q defects at intermediate follow-up	76	63 (83%)	0 (0%)	10 (83%)	11 (79%)	29 (85%)	13 (87%)	0.41
Subsegmental, total	55	189	0	26	29	84	50	
Subsegmental, ratio		3.4	0	3.7	3.2	3.2	3.8	
Segmental, total	38	95	0	14	23	48	10	
Mismatched Q defects at short-term follow-up	147	83 (56%)	1 (100%)	16 (44%)	16 (76%)	33 (49%)	17 (77%)	0.014^R^
Subsegmental, total	82	171	2	27	33	74	35	
Subsegmental, ratio		2.1	2	1.8	2.1	2.2	2.1	
Segmental, total	7	11	0	4	4	3	0	
Mismatched Q defects at intermediate follow-up	77	36 (47%)	0 (0%)	5 (38%)	6 (43%)	18 (53%)	7 (47%)	0.86
Subsegmental, total	31	55	0	5	9	27	14	
Subsegmental, ratio		1.8	0	1.7	2.3	1.6	2.0	
Segmental, total	7	12	0	6	1	5	0	
Matched V/Q defects at short-term follow-up	147	59 (40%)	1 (100%)	11 (31%)	9 (43%)	27 (40%)	11 (50%)	0.43
Subsegmental, total	55	104	2	18	19	47	18	
Subsegmental, ratio		1.9	2	1.8	2.4	1.8	1.8	
Segmental, total	12	19	0	3	1	8	7	
Matched V/Q defects at intermediate follow-up	76	38 (50%)	0 (0%)	6 (50%)	6 (43%)	20 (59%)	6 (40%)	0.60
Subsegmental, total	29	64	0	17	7	27	13	
Subsegmental, ratio		2.2	0	3.4	1.4	1.9	2.6	
Segmental, total	12	22	0	7	4	10	1	
Inversely mismatched V defects at short-term follow-up	146	94 (64%)	0 (0%)	16 (44%)	16 (76%)	45 (68%)	17 (77)	0.020^R^
Subsegmental, total	74	230	0	38	40	105	47	
Subsegmental, ratio		3.1	0	2.5	3.6	3.2	3.1	
Segmental, total	49	133	0	9	29	74	21	
Inversely mismatched V defects at intermediate follow-up	76	42 (55%)	0 (0%)	4 (33%)	7 (50%)	23 (68%)	8 (53%)	0.19
Subsegmental, total	29	70	0	4	13	30	23	
Subsegmental, ratio		2.4	0	1.3	2.6	2.1	3.3	
Segmental, total	25	61	0	1	18	33	9	
Normal V/Q scan^*^								
short-term follow-up	147	44 (30%)	0 (0%)	19 (53%)	5 (24%)	19 (28%)	1 (5%)	0.001^D^
intermediate follow-up	76	22 (29%)	1 (100%)	6 (50%)	4 (29%)	8 (34%)	3 (20%)	0.21

Data are expressed as *n* (%), total sum of defects or ratio between number of subsegmental defects and number of patients with subsegmental defects

Q: perfusion, V: ventilation, Mismatched Q defects: perfusion defects, but normal ventilation in the area, inversely mismatched V defects: ventilation defects, but normal perfusion in the area

*No ventilatory or vascular abnormality

D) Mild differs from severe and critical; R) No differences between severity groups after correction for multiple analyses; S) Mild differs from moderate

### Factors associated with reduced D_L,COc_ at short-term follow-up

The extent of D_L,COc_ reduction was associated with the extent of GGO and pulmonary fibrosis on HRCT, as well as the number of matched and inversely mismatched ventilatory defects, but not with mismatched perfusion defects on V/Q SPECT (Table 4). Reduced D_L,COc_ was associated with clinical severity group but not with age or sex. Similarly, GGO > 25% and pulmonary fibrosis was associated with clinical severity group and age, whereas only pulmonary fibrosis was also associated with sex (Online Resource 3). No associations were observed with defects on V/Q SPECT (Online Resource 3).

**Table 4:** Association between CAT score, V/Q SPECT defects or HRCT findings with haemoglobin-corrected pulmonary diffusing capacity for carbon monoxide (D_L,COc_ %predicted) in patients at short-term follow-up after COVID-19 (*n* = 151)

	B	95% CI	*P*-value
Clinical findings			
CAT score	−0.4	−0.7; 0.2	0.060
HRCT findings			
GGO extent	−1.3	−1.8; −0.9	< 0.001
PF extent	−2.3	−3.1; −1.5	< 0.001
SPECT findings			
Number of V/Q defects	−1.4	−2.2; −0.7	< 0.001
Number of mismatched Q defects	−0.1	−2.1; 1.9	0.93
Number of matched V/Q defects	−3.3	−5.2; −1.4	< 0.001
Number of inversely mismatched defects	−1.3	−2.3; −0.3	0.009

CAT score: chronic obstructive pulmonary disease assessment test, GGO: ground-glass opacities, HRCT: high-resolution computed tomography, PF: pulmonary fibrosis, SPECT: single photon emission computed tomography, V/Q: ventilation-perfusion

### Nasal clearance and blood biochemistry

In most individuals, nasal clearance was normal at both short-term and intermediate follow-up (Online Resources 4 and 5), with no differences observed between the two follow-up periods (data not shown). Blood biochemistry findings are presented in Online Resources 6 and 7. Clinical severity was associated with haemoglobin, ferritin, and creatinine levels at both short-term and intermediate follow-up. Associations with CRP, D-dimer, and ALAT were observed only at short-term follow-up.

## Discussion

In the present study, we observed that a reduced D_L,COc_ at short-term follow-up (approximately 6 months) was associated with a severity-dependent restrictive lung function pattern, characterised by reductions in FVC, TLC, and RV, as well as concomitant GGO and fibrosis on HRCT and ventilatory disturbances on V/Q SPECT. By intermediate follow-up at approximately 12 months, D_L,COc_ had increased alongside an improvement in FVC, and was no longer associated with initial COVID-19 severity. However, reductions in RV, as well as the persistence of GGO and fibrosis on HRCT and ventilatory disturbances on V/Q SPECT, remained at intermediate follow-up and appeared to be dependent on initial clinical severity. Nasal clearance was normal in most patients and showed no significant difference between follow-up times and will not be discussed further here.

The prevalence and extent of D_L,COc_ reduction at short-term follow-up were found to depend on initial clinical severity, which is consistent with previous studies conducted with up to 12-month follow-up from China, Chile, Germany, Mexico, Denmark, and the United States [6, 8, 12, 37–41]. The reduction in D_L,COc_ has in earlier studies been found to be unspecific as no consistent pattern in contribution from membrane diffusing capacity (D_M_), depending on thickness and area of the alveolar-capillary membrane available for gas exchange and pulmonary capillary blood volume (V_C_) exists [5]. The exact prevalence estimates are challenging to compare across countries due to the varying impact of the COVID-19 pandemic on different regions, largely because of differences in preventive, diagnostic, and therapeutic strategies, as well as hospitalisation and ICU admission thresholds. In the present study, findings at intermediate follow-up demonstrate that D_L,COc_ and FVC improved and became unrelated to initial clinical severity—a finding not previously reported. Nonetheless, D_L,COc_ has also been reported to gradually improve up to 12 months after discharge in studies from China and Mexico, but still remains low in most patients [8, 40], and seems to improve further at long-term follow-up, i.e. at 28 months according to a recent study from France [42]. However, the persistent reduction in RV related to the initial clinical severity is a novel finding and may represent a more specific marker of post-COVID restrictive lung disease.

In terms of HRCT findings, GGO was the most common abnormality observed in 60% of the patients at short-term follow-up and 88% of the patients that were assessed at intermediate follow-up. This aligns well with previous studies and their reported associations with clinical severity at different follow-up times [3, 6, 8, 11–13]. The presence of GGO indicates localised infection, inflammation, or fluid accumulation in the interstitial or alveolar spaces, none of which are mutually exclusive [43]. These findings likely reflect residual changes from the acute infection. Accordingly, the extent of GGO following COVID-19 has previously been associated with peak HRCT pneumonia scores during hospitalisation, and GGO scores have been reported to gradually decrease over the first 12 months of follow-up [4, 7, 8, 13, 39, 40, 44]. This likely provides a mechanistic link to reduced D_L,COc_, as supported by our observation that reduced D_L,COc_ was associated with the extent of GGO. Nevertheless, while GGO, along with other HRCT abnormalities such as fibrosis, largely persists at one year follow-up and remains associated with initial disease severity, gradually recovery of D_L,COc_ is observed, such that its association with initial disease severity disappears. This is in accordance with our working hypothesis that a dissociation between the decline in diffusing capacity and structural changes on HRCT occurs beyond the short-term follow-up period, but the underlying mechanisms that cause this phenomenon remain elusive.

This study provides updated data from our previous smaller cohort [41]. The increase in sample size reinforces the previous findings from the smaller cohort regarding D_L,COc,_ HRCT and V/Q SPECT. Similar proportions of participants were found with reduced D_L,COc_, associated with GGO on HRCT, but not with mismatched perfusion defects on V/Q SPECT in both cohorts. Also, GGO was the most common finding on HRCT. According to V/Q SPECT, 95% had V/Q defects in the smaller cohort with slightly fever in the larger cohort (87%). Likewise, both cohorts found most participants to be represented with ventilatory abnormalities, however ventilatory abnormalities were more frequent in the smaller cohort. Additionally, 66% of the smaller cohort and 56% of the larger cohort were represented with mismatched perfusion defects, 40% were represented with matched V/Q defects in both cohorts, and inversely mismatched ventilatory defects were found in 75% of the smaller cohort and 64% of the larger cohort. Additionally, a higher proportion of participants were represented with normal V/Q scans in the larger cohort. In both cohorts the number of matched but not mismatch defects on the V/Q SPECT were found to be associated with the reduced D_LCOc_.

The V/Q SPECT findings at intermediate follow-up demonstrated minor alterations relative to short-term follow-up. A slightly smaller proportion of participants were represented with at least one type of V/Q abnormality at intermediate follow-up, being slightly higher in moderate, severe and critical compared to the mild group at short-term follow-up, with a more equal distribution of V/Q abnormalities between groups at intermediate follow-up. Mismatched perfusion defects (47%) and inversely mismatched ventilatory defects (55%) were slightly lower and matched V/Q defects were slightly higher (50%) at intermediate follow-up. The proportion of normal V/Q scans at short-term and intermediate follow-up were the same (30% at short-term and 29% at intermediate).

Anticoagulation treatment was administered routinely as systematic prophylaxis during admission. Only three received therapeutic doses which is in accordance with the low level of vascular abnormalities. The prophylaxis in those with highest disease severity most likely reduced the COVID-19 related thrombo-embolic complications.

This study has several limitations which may affect the generalisability of our findings. Firstly, although all patients discharged from Rigshospitalet were invited to participate, certain groups were excluded from the analysis. These include those patients residing in care facilities, and others at high risk of severe COVID-19, who may experience more pronounced long-term complications. Conversely, individuals experiencing symptoms potentially linked to COVID-19 may have been more motivated to enrol in the study, potentially introducing selection bias. On a related note, there was only one asymptomatic patient in this cohort. Furthermore, among the non-hospitalised participants, there was an overrepresentation of healthcare workers. Additionally, while we have ruled out primary pulmonary perfusion disturbances as the cause of reduced D_L,COc_ post-COVID-19, we cannot ascertain the clinical significance of the abnormalities detected on V/Q SPECT, nor their potential impact on long-term prognosis. Additionally, only participants indicative of COVID-19 sequelae at short-term follow-up were referred to re-assessment at intermediate follow-up, and not all examinations were performed on all participants at each time point. Also, the findings for both D_L,COc_, HRCT and V/Q SPECT subject to be influenced by pre-existing pulmonary abnormalities, however only seven participants were represented with pre-existing pulmonary abnormalities, thus the comorbidities of the participants might have influenced the results to a lesser extent. At this stage, our findings do not provide prognostic insights into the observed clinical, physiological, or imaging abnormalities and therefore cannot directly guide clinical decision-making. Finally, the intermediate follow-up strategy may have introduced further selection bias, as individuals without significant abnormalities were not invited for subsequent follow-up assessments.

Regarding the clinical implications of our findings for practice and future research, this study highlights that a significant proportion of individuals infected with SARS-CoV-2 display both structural and functional abnormalities in the lung parenchyma, often accompanied by restrictive lung disease and reduced D_L,COc_. Although such restrictive lung disease-like changes were previously considered largely irreversible, the present findings suggest that they resolve over time in many patients. While there are no established treatment options proven effective for the associated post-COVID reductions in D_L,COc_ during short-term follow-up, intervention may not be necessary in most instances as D_L,COc_ recover spontaneously. Nevertheless, it remains unclear whether residual structural abnormalities, such as reduced RV and ventilatory disturbances observed on V/Q SPECT, are associated with persistent symptoms or increase the risk of developing long-term lung disease. Further investigation is needed to elucidate these relationships.

In conclusion, the post-COVID severity-dependent reduction in D_L,COc_ at approximately six months, associated with restrictive lung function, GGO and fibrosis on HRCT, and ventilatory disturbances on V/Q SPECT shows spontaneous recovery by intermediate follow-up at approximately 12 months. However, the restrictive ventilatory disturbances and associated morphological changes largely persist. Further investigation is warranted to determine the long-term implications of these residual structural and ventilatory changes and to explore targeted interventions or rehabilitation strategies to improve outcomes in affected individuals.

## Supplementary Information

Below is the link to the electronic supplementary material.

Supplementary Material 1

## Data Availability

The data underlying our findings can be shared upon reasonable request directed to the corresponding author.
