# Supplementary material for: Relation of pulmonary diffusing capacity changes to HRCT chest and V/Q SPECT findings at short-term and intermediate follow-up after COVID-19: a prospective cohort study (The secure study)
Source: Eur J Nucl Med Mol Imaging. 2025 Oct 30;53(3):2178–89. doi: 10.1007/s00259-025-07598-0 (PMC12860846; doi:10.1007/s00259-025-07598-0)
Supplement: Supplementary file 1 — Supplementary Material 1 [file 259_2025_7598_MOESM1_ESM.docx]

**Online Resource 1:** Lung function outcome short-term and intermediate follow-up after COVID-19 (n=153;38) and differences between severity groups

| **Parameter** | **n** | **All (n=153;38)** | **Asymptomatic (n=1;0)** | **Mild (n=38;5)** | **Moderate (n=23;7)** | **Severe (n=69;17)** | **Critical (n=22;9)** | **Between group p-value** | |
| --- | --- | --- | --- | --- | --- | --- | --- | --- | --- |
| FEV_1_ %P |  |  |  |  |  |  |  |  | |
| short-term follow-up | 153 | 108.0 [96.0; 121.1] | 103.1 | 112.0 [101.0; 119.2] | 108.7 [94.5; 121.7] | 109.3 [93.4; 124.5] | 97.8 [90.6; 119.2] | 0.63 | |
| intermediate follow-up | 38 | 98.7 [81.4; 109.9] | - | 96.2 [81.4; 101.4] | 85.0 [71.5; 123.2] | 94.4 [83.6; 109.4] | 101.9 [98.7; 109.9] | 0.68 | |
| FVC %P |  |  |  |  |  |  |  |  | |
| short-term follow-up | 153 | 113.7 [97.6; 123.5] | 125.8 | 120.3 [109.6; 127.5] | 114.0 [97.2; 124.0] | 113.7 [95.6; 121.6] | 102.7 [90.9; 119.5] | 0.017^D^ | |
| intermediate follow-up | 38 | 99.1 [90.1: 109.1] | - | 108.3 [90.3; 115.1] | 95.7 [75.9; 129.9] | 98.0 [90.1; 100.5] | 101.3 [98.8; 109.1] | 0.64 | |
| FEV_1_/FVC %P |  |  |  |  |  |  |  |  | |
| short-term follow-up | 153 | 101.3 [96.0; 106.5] | 87.3 | 97.8 [92.6; 103.2] | 100.1 [95.9; 105.2] | 103.8 [98.0; 108.8] | 105.3 [99.5; 108.9] | <0.001^D^ | |
| intermediate follow-up | 38 | 100.3 [92.1; 107.4] | - | 95.9 [92.1; 99.7] | 95.9 [85.7; 100.4] | 104.3 [98.8; 110.5] | 104.7 [100.1; 107.4] | 0.14 | |
| TLC %P |  |  |  |  |  |  |  |  | |
| short-term follow-up | 150 | 101.8 [90.7; 110.3] | 117.0 | 109.9 [101.4; 116.3] | 106.8 [91.1; 110.3] | 99.8 [90.0; 106.5] | 86.9 [82.1; 101.8] | <0.001^E^ | |
| intermediate follow-up | 28 | 86.1 [79.3; 94.2] | - | 89.2 | 96.1 [83.0; 103.4] | 82.8 [80.2; 93.1] | 85.2 [78.4; 89.8] | 0.65 | |
| RV %P |  |  |  |  |  |  |  |  | |
| short-term follow-up | 150 | 88.7 [78.0; 102.1] | 108.6 | 94.8 [79.6; 106.8] | 88.2 [82.1; 100.6] | 89.1 [78.8; 102.1] | 70.5 [63.2; 81.7] | <0.001^F^ | |
| intermediate follow-up | 28 | 71.3 [64.1; 85.5] | - | 99.9 | 85.6 [81.0; 107.2] | 72.8 [67.5; 85.3] | 63.5 [59.0; 54.5] | 0.003^G^ | |
| RV/TLC %P |  |  |  |  |  |  |  |  | |
| short-term follow-up | 150 | 83.6 [75.6; 92.0] | 90.2 | 83.9 [75.6; 94.4] | 85.7 [78.8; 89.7] | 83.7 [78.0; 98.0] | 72.8 [70.2; 84.7] | 0.041^H^ | |
| intermediate follow-up | 28 | 78.1 [71.0; 94.0] | - | 103.9 | 90.9 [86.2; 93.3] | 82.6 [74.3; 96.6] | 67.9 [64.3; 74.3] | 0.003^G^ | |
| D_L,COc_ %P |  |  |  |  |  |  |  |  | |
| short-term follow-up | 151 | 78.7 [71.6; 88.9] | 76.7 | 83.3 [77.6; 99.4] | 85.1 [73.6; 94.0] | 78.2 [70.9; 85.1] | 66.1 [56.8; 76.4] | <0.001^B^ | |
| intermediate follow-up | 86 | 75.7 [66.9; 84.6] | 72.7 | 80.1 [75.2; 83.6] | 73.2 [62.7; 82.7] | 78.8 [69.3; 85.9] | 67.7 [62.8; 74.5] | 0.10 | |
| K_CO_ %P |  |  |  |  |  |  |  |  | |
| short-term follow-up | 151 | 91.8 [83.1; 102.7] | 75.0 | 92.9 [83.3; 104.1] | 91.8 [83.3; 103.2] | 94.8 [83.2; 103.8] | 89.9 [78.5; 95.9] | 0.40 | |
| intermediate follow-up | 85 | 89.4 [83.2; 99.1] | 76.0 | 87.5 [82.9; 95.2] | 88.3 [82.2; 99.0] | 91.3 [84.1; 100.2] | 89.5 [83.4; 100.7] | 0.63 | |
| **Ventilation at short-term follow-up (no.)** | 152 |  |  |  |  |  |  |  | |
| Restriction |  | 12 (13%) | 0 (0%) | 1 (3%) | 1 (4%) | 10 (14%) | 8 (38%) | 0.002^F^ | |
| Obstruction |  | 6 (4%) | 0 (0%) | 0 (0%) | 1 (4%) | 4 (6%) | 1 (5%) | 0.45 | |
| Both restriction and obstruction |  | 0 (0%) | 0 (0%) | 0 (0%) | 0 (0%) | 0 (0%) | 0 (0%) | - | |
| **Ventilation at intermediate follow-up** | 38 |  |  |  |  |  |  |  | |
| Restriction (no.) |  | 12 (32%) | - | 0 (0%) | 1 (14%) | 8 (44%) | 3 (33%) | 0.31 | |
| Obstruction (no.) |  | 4 (11%) | - | 0 (0%) | 2 (29%) | 2 (11%) | 0 (0%) | 0.27 | |
| Both restriction and obstruction (no.) |  | 0 (0%) | - | 0 (0%) | 0 (0%) | 0 (0%) | 0 (0%) | - | |
| **Diffusing capacity at short-term follow-up (no.)** |  |  |  |  |  |  |  |  | |
| Reduced D_L,COc_ | 151 | 62 (41%) | 1 (100%) | 9 (24%) | 7 (30%) | 31 (46%) | 14 (67%) | 0.006^P^ | |
| D_L,COc_ >LLN |  |  | 0 (0%) | 29 (76%) | 16 (70%) | 37 (54%) | 7 (33%) |  | |
| D_L,COc_ 60%P-LLN |  |  | 1 (100%) | 9 (24%) | 4 (17%) | 22 (32%) | 5 (24%) |  | |
| D_L,COc_ <60%P |  |  | 0 (0%) | 0 (0%) | 3 (13%) | 9 (13%) | 9 (43%) |  | |
| **Diffusing capacity at intermediate follow-up (no.)** |  |  |  |  |  |  |  |  | |
| Reduced D_L,COc_ | 85 | 47 (55%) | 1 (100%) | 7 (50%) | 8 (67%) | 18 (45%) | 13 (72%) | 0.23 | |
| D_L,COc_ >LLN |  | 38 (45%) | 0 (0%) | 7 (50%) | 4 (33%) | 22 (55%) | 5 (28%) |  | |
| D_L,COc_ 60%P-LLN |  | 38 (45%) | 1 (100%) | 6 (43%) | 5 (42%) | 15 (38%) | 11 (61%) |  | |
| D_L,COc_ <60%P |  | 9 (11%) | 0 (0%) | 1 (7%) | 3 (25%) | 3 (8%) | 2 (11%) |  | |
| **Both ventilation and diffusing capacity at short-term follow-up (no.)** | 151 |  |  |  |  |  |  | 0.001 | |
| Normal |  | 84 (56%) | 0 (0%) | 29 (76%) | 16 (70%) | 33 (49%) | 6 (29%) | 0.001^Q^ | |
| Restriction + low D_L,COc_ |  | 16 (11%) | 0 (0%) | 1 (3%) | 1 (4%) | 6 (9%) | 8 (38%) | 0.001^F^ | |
| Restriction + normal D_L,COc_ |  | 4 (3%) | 0 (0%) | 0 (0%) | 0 (0%) | 4 (6%) | 0 (0%) | 0.33 | |
| Obstruction + low D_L,COc_ |  | 5 (3%) | 0 (0%) | 0 (0%) | 1 (4%) | 4 (6%) | 0 (0%) | 0.43 | |
| Obstruction + normal D_L,COc_ |  | 1 (1%) | 0 (0%) | 0 (0%) | 0 (0%) | 0 (0%) | 1 (5%) | 0.15 | |
| Low D_L,COc_ only |  | 41 (27%) | 1 (100%) | 8 (21%) | 5 (22%) | 21 (31%) | 6 (29%) | 0.43 | |
| **Both ventilation and diffusing capacity at intermediate follow-up (no.)** | 35 |  |  |  |  |  |  | 0.68 | |
| Normal |  | 10 (29%) | - | 2 (67%) | 3 (43%) | 3 (19%) | 2 (22%) |  | |
| Restriction + low D_L,COc_ |  | 9 (26%) | - | 0 (0%) | 1 (14%) | 5 (31%) | 3 (33%) |  | |
| Restriction + normal D_L,COc_ |  | 2 (6%) | - | 0 (0%) | 0 (0%) | 2 (13%) | 0 (0%) |  | |
| Obstruction + low D_L,COc_ |  | 4 (11%) | - | 0 (0%) | 2 (29%) | 2 (13%) | 0 (0%) |  | |
| Obstruction + normal D_L,COc_ |  | - | - | - | - | - | - |  | |
| Low D_L,COc_ only |  | 10 (29%) | - | 1 (33%) | 1 (14%) | 4 (25%) | 4 (44%) |  | |
| Data are expressed as n (%), mean (SD) or median [interquartile range] as appropriate.  Abbreviations: D_L,COc_: Haemoglobin corrected diffusing capacity for carbon monoxide, FEV_1_: Forced expiratory volume in the first second, FVC: forced vital capacity , K_CO_: diffusion coefficient for carbon monoxide, LLN: lower limit of normal, P: predicted, RV: residual volume, TLC: total lung capacity. B) Critical differs from mild, moderate, and severe and mild differs from severe; D) Mild differs from severe and critical; E) Mild differs from severe and critical and moderate differs from severe; F) Critical differs from mild, moderate, and severe; G) Critical differs from moderate and severe; H) Critical differs from mild and severe; P) Mild differs from critical; Q) Mild differs from severe and critical and moderate differs from critical. | | | | | | | | |  |

**Online Resource 2:** Difference in lung function between short-term and intermediate follow-up after COVID-19 for those (n=85) with abnormal lung function

|  | n | Short-term follow-up | Intermediate follow-up | Difference in mean (95% CI) | p-value |
| --- | --- | --- | --- | --- | --- |
| FEV_1_ %P | 38 | 90.2 [78.0; 103.1] | 98.7 [81.4; 109.9] | 4.62 (1.77; 7.33) | 0.001 |
| FVC %P | 38 | 92.5 [86.0; 102.8] | 99.1 [90.1: 109.1] | 4.53 (1.52; 7.37) | 0.002 |
| FEV_1_/FVC %P | 38 | 102.2 [94.6; 109.7] | 100.3 [92.1; 107.4] | -1.20 (-3.3; 0.55) | 0.22 |
| TLC %P | 28 | 81.9 [76.3; 90.7] | 86.1 [79.3; 94.2] | 2.34 (-0.24; 5.06) | 0.079 |
| RV %P | 28 | 72.0 [63.5; 85.8] | 71.3 [64.1; 85.5] | -2.84 (-8.08; 1.57) | 0.25 |
| RV/TLC %P | 28 | 83.6 [74.6; 97.6] | 78.1 [71.0; 94.0] | -1.71 (-6.00; 2.26) | 0.42 |
| D_L,COc_ %P | 85 | 74.9 [62.7; 80.7] | 75.7 [66.9; 84.6] | 3.09 (1.19; 5.06) | 0.002 |
| K_CO_ %P | 85 | 88.3 [80.0; 96.5] | 89.4 [83.2; 99.1] | 1.97 (-0.25; 4.25) | 0.083 |
| Data are expressed as median [interquartile range].  Abbreviations: D_L,COc_: Haemoglobin corrected diffusing capacity of carbon monoxide, FEV_1_: Forced expiratory volume in the first second, FVC: forced vital capacity, K_CO_: diffusion coefficient for carbon monoxide, P: predicted, RV: residual volume, TLC: total lung capacity | | | | | |

**Online Resource 3:** Association between V/Q defects, HRCT findings (ground glass opacities (GGO) or sign of fibrosis (PF)) or haemoglobin-corrected pulmonary diffusing capacity for carbon monoxide (D_L,COc_ %predicted) with severity of disease, age, and sex in patient’s short-term follow-up after COVID-19 (n=151)

|  | ***n*** | **Odds ratio** | **95% CI** | **P-value** |
| --- | --- | --- | --- | --- |
| Mismatched Q defects | 147 |  |  |  |
| Severity group |  | 1.30 | 0.90; 1.86 | 0.16 |
| Age in years |  | 0.98 | 0.96; 1.01 | 0.22 |
| Sex, female |  | 0.83 | 0.41; 1.70 | 0.61 |
| Matched V/Q defects | 147 |  |  |  |
| Severity group |  | 1.04 | 0.72; 1.50 | 0.84 |
| Age in years |  | 1.03 | 1.01; 1.06 | 0.014 |
| Sex, f |  | 1.23 | 0.59; 2.56 | 0.58 |
| GGO >25% | 144 |  |  |  |
| Severity group |  | 7.77 | 3.11; 19.40 | <0.001 |
| Age in years |  | 1.05 | 1.01; 1.09 | 0.012 |
| Sex, female |  | 0.48 | 0.18; 1.33 | 0.16 |
| PF | 144 |  |  |  |
| Severity group |  | 2.67 | 1.62; 4.36 | <0.001 |
| Age in years |  | 1.04 | 1.01; 1.07 | 0.005 |
| Sex, female |  | 0.40 | 0.17; 0.91 | 0.030 |
| Reduced D_L,COc_ | 151 |  |  |  |
| Severity group |  | 1.61 | 1.10; 2.36 | 0.014 |
| Age in years |  | 1.01 | 0.99; 1.04 | 0.28 |
| Sex, female |  | 1.19 | 0.57; 2.47 | 0.64 |

Abbreviations: HRCT: high-resolution computed tomography, Q: perfusion, V: ventilation.

**Online Resource 4:** Nasal clearance in patients at short-term and intermediate follow-up after COVID-19 (n=139;11) and differences between severity groups

| **Parameter** | **n** | **All (n=139;11)** | **Asymptomatic (n=1;0)** | **Mild (n=37;1)** | **Moderate (n=20;4)** | **Severe (n=61;5)** | **Critical (n=20;1)** | **Between group p-value** |
| --- | --- | --- | --- | --- | --- | --- | --- | --- |
| Nasal saccharin clearance time, min |  |  |  |  |  |  |  |  |
| short-term follow-up | 139 | 9 [7; 12] | 10 | 9 [9; 11] | 10 [6; 14] | 10 [7; 11] | 10 [8; 11] | 0.97 |
| intermediate follow-up | 11 | 12 [10; 23] | - | 10 | 11 [8; 22] | 12 [12; 15] | 23 | 0.56 |
| Nasal saccharin clearance at short-term follow-up (no.) | 139 |  |  |  |  |  |  | 0.023^T^ |
| Normal |  | 133 (96%) | 1 (100%) | 37 (100%) | 16 (80%) | 59 (97%) | 20 (100%) |  |
| Low |  | 6 (4%) | 0 (0%) | 0 (0%) | 4 (20%) | 2 (3%) | 0 (0%) |  |
| Nasal saccharin clearance at intermediate follow-up (no.) | 11 |  |  |  |  |  |  | 0.58 |
| Normal |  | 8 (73%) | - | 1 (100%) | 3 (75%) | 4 (80%) | 0 (0%) |  |
| Low |  | 3 (27%) | - | 0 (0%) | 1 (25%) | 1 (20%) | 1 (100%) |  |
| Data are expressed as n (%), mean (SD) or median [interquartile range] as appropriate.  T) Moderate differs from mild, severe, and critical | | | | | | | | |

**Online Resource 5:** Difference in nasal clearance between short-term and intermediate follow-up after COVID-19 for those (n=5) with abnormal nasal clearance at short-term follow-up and intermediate follow-up

|  | short-term follow-up | intermediate follow-up | Difference (95% CI) | p-value |
| --- | --- | --- | --- | --- |
| Nasal saccharin clearance time, min | 30 [26; 31] | 10 [10; 30] | -17 (-37; 4) | 0.10 |
| Data are expressed as median [interquartile range] | | | | |

**Online Resource 6:** Blood samples in patients at short-term and intermediate follow-up after COVID-19 (n=148;108) and differences between severity groups

| **Parameter** | **n** | **All (n=148;108)** | **Asymptomatic (n=1;1)** | **Mild (n=36;18)** | **Moderate (n=23;17)** | **Severe (n=66;53)** | **Critical (n=22;19)** | **Between group p-value** |
| --- | --- | --- | --- | --- | --- | --- | --- | --- |
| Haemoglobin, mmol/L |  |  |  |  |  |  |  |  |
| short-term follow-up | 148 | 8.8 [8.2; 9.2] | 8.2 | 8.4 [8.1; 9.1] | 8.6 [7.7; 8.9] | 9.1 [8.5; 9.5] | 8.6 [8.3; 9.1] | 0.002^U^ |
| intermediate follow-up | 108 | 8.8 [8.1; 9.4] | 7.8 | 8.4 [7.9; 8.5] | 8.5 [8.0; 9.2] | 9.1 [8.6; 9.6] | 8.8 [8.0; 9.5] | 0.001^V^ |
| Ferritin, µg/L |  |  |  |  |  |  |  |  |
| short-term follow-up | 142 | 103 [57; 181] | 64 | 73 [44; 111] | 85 [57; 133] | 126 [79; 220] | 111 [55; 201] | 0.023^V^ |
| intermediate follow-up | 99 | 107 [51; 197] | 50 | 38 [28; 92] | 72 [49; 102] | 150 [86; 232] | 118 [74; 234] | <0.001^E^ |
| Platelets, 10^9^/L |  |  |  |  |  |  |  |  |
| short-term follow-up | 146 | 242 [208; 283] | 249 | 244 [230; 268] | 250 [220; 327] | 220 [199; 279] | 267 [220; 294] | 0.19 |
| intermediate follow-up | 107 | 231 [201; 275] | 231 | 235 [218; 295] | 266 [211; 301] | 228 [197; 261] | 214 [194; 266] | 0.25 |
| Leucocytes, 10^9^/L |  |  |  |  |  |  |  |  |
| short-term follow-up | 148 | 7.0 [5.7; 8.4] | 5.9 | 7.3 [6.1; 8.3] | 7.9 [5.4; 9.1] | 6.9 [5.6; 8.0] | 6.9 [5.9; 8.5] | 0.88 |
| intermediate follow-up | 108 | 6.6 [5.6; 8.1] | 5.5 | 6.6 [5.9; 7.3] | 5.8 [7.4; 8.7] | 6.6 [5.6; 8.2] | 6.2 [5.3; 8.3] | 0.71 |
| Neutrophiles, 10^9^/L |  |  |  |  |  |  |  |  |
| short-term follow-up | 141 | 4.1 [3.0; 5.1] | 3.0 | 4.4 [3.6; 5.3] | 4.6 [2.2; 5.9] | 4.0 [3.0; 5.0] | 3.8 [3.0; 4.5] | 0.73 |
| intermediate follow-up | 102 | 4.0 [3.1; 4.8] | 3.5 | 4.2 [3.0; 4.5] | 4.3 [3.0; 4.8] | 4.0 [3.2; 4.8] | 3.6 [3.1; 5.0] | 0.97 |
| C-reactive protein, mg/L |  |  |  |  |  |  |  |  |
| short-term follow-up | 146 | 1 [1; 3] | 1 | 1 [1; 2] | 2 [1; 6] | 2 [1; 3] | 2 [1; 5] | 0.021^X^ |
| intermediate follow-up | 105 | 2 [1; 3] | 1 | 1 [1; 2] | 3 [1; 4] | 1 [1; 3] | 2 [1; 4] | 0.11 |
| D-dimer, mg/L |  |  |  |  |  |  |  |  |
| short-term follow-up | 135 | 0.3 [0.3; 0.4] | 0.3 | 0.3 [0.3; 0.3] | 0.3 [0.3; 0.7] | 0.3 [0.3; 0.4] | 0.3 [0.3; 0.5] | 0.040^S^ |
| intermediate follow-up | 96 | 0.3 [0.3; 0.4] | 0.6 | 0.3 [0.3; 0.3] | 0.3 [0.3; 0.6] | 0.3 [0.3; 0.4] | 0.3 [0.3; 0.4] | 0.26 |
| ALAT, U/L |  |  |  |  |  |  |  |  |
| short-term follow-up | 146 | 23 [17; 31] | 16 | 19 [16; 27] | 20 [16; 34] | 25 [21; 34] | 23 [17; 28] | 0.050 |
| intermediate follow-up | 105 | 22 [19; 32] | 22 | 21 [17; 22] | 20 [17; 34] | 24 [20; 34] | 25 [19; 33] | 0.080 |
| Creatinine, µmol/L |  |  |  |  |  |  |  |  |
| short-term follow-up | 148 | 79 [65; 91] | 71 | 70 [58; 80] | 74 [61; 91] | 83 [66; 94] | 85 [67; 100] | 0.008^D^ |
| intermediate follow-up | 107 | 77 [64; 90] | 66 | 64 [58; 74] | 81 [62; 87] | 82 [70; 90] | 79 [71; 93] | 0.017^D^ |
| Data are expressed as median [interquartile range].  Abbreviations: ALAT=Alanine transaminase  D) Mild differs from severe and critical; E) Mild differs from severe and critical and moderate differs from severe; S) Mild differs from moderate; U) Severe differs from mild and moderate; V) Severe differs from mild; X) Mild differs from moderate and critical | | | | | | | | |

**Online Resource 7:** Difference in blood samples between short-term and intermediate follow-up after COVID-19 for those (n=107) assessed at both short-term and intermediate follow-up

|  | n | short-term follow-up | intermediate follow-up | Difference in mean (95% CI) | p-value |
| --- | --- | --- | --- | --- | --- |
| Haemoglobin, mmol/L | 107 | 8.7 [8.1; 9.1] | 8.8 [8.1; 9.4] | 0.08 (-0.01; 0.07) | 0.07 |
| Ferritin, µg/L | 94 | 98 [54; 197] | 107 [51; 197] | -1.8 (-18.7; 10.6) | 0.81 |
| Platelets, 10^9^/L | 104 | 245 [208; 278] | 232 [201; 275] | -5.5 (-13.5; 4.1) | 0.22 |
| Leucocytes, 10^9^/L | 107 | 7.0 [5.6; 8.3] | 6.6 [5.6; 8.1] | -4.0 (-14.7; 0.04) | 0.29 |
| Neutrophiles, 10^9^/L | 98 | 3.9 [3.0; 5.1] | 4.0 [3.1; 4.6] | 4.6 (-0.2; 17.7) | 0.32 |
| C-reactive protein, mg/L | 103 | 1 [1; 3] | 2 [1; 3] | -2.0 (-7.6; 0.5) | 0.35 |
| D-dimer, mg/L | 87 | 0.3 [0.3; 0.4] | 0.3 [0.3; 0.4] | -0.09 (-0.29; 0.01) | 0.25 |
| ALAT, U/L | 102 | 23 [17; 30] | 22 [19; 32] | 0.7 (-1.1; 2.6) | 0.43 |
| Creatinine, µmol/L | 106 | 80 [66; 92] | 78 [64; 90] | -2.5 (-4.8; -0.2) | 0.04 |
| Data are expressed as median [interquartile range].  Abbreviations: ALAT=Alanine transaminase | | | | | |
